# Prediction of deciduous teeth eruption in Brazilian children: A cross-sectional study nested in a prospective birth cohort (BRISA)

**DOI:** 10.1186/s12903-023-03823-0

**Published:** 2024-01-09

**Authors:** Rafiza Felix Marão Martins, Alcione Miranda dos Santos, Maria da Conceição Pereira Saraiva, Cecília Cláudia Costa Ribeiro, Cláudia Maria Coelho Alves, Antônio Augusto Moura da Silva, Heloisa Betiol, Marco Antonio Barbieri, Erika Barbara Abreu Fonseca Thomaz

**Affiliations:** 1 Federal University of Maranhao, São Luís, Brazil; 2 Ribeirão Preto Medical School, University of São Paulo, Ribeirão Preto, Brazil

**Keywords:** Tooth eruption, Estimation technics, Growth charts

## Abstract

**Background:**

Dental eruption is part of a set of children´s somatic growth phenomena. The worldwide accepted human dental eruption chronology is still based on a small sample of European children. However, evidence points to some population variations with the eruption at least two months later in low-income countries, and local standards may be useful. So, this study aimed to predict deciduous teeth eruption from 12 months of age in a Brazilian infant population.

**Methods:**

We developed a cross-sectional study nested in four prospective cohorts – the Brazilian Ribeirão Preto and São Luís Cohort Study (BRISA) – in a sample of 3,733 children aged 12 to 36 months old, corrected by gestational age. We made a reference curve with the number of teeth erupted by age using the Generalized Additive Models for location, scale, and shape (GAMLSS) technique. The explanatory variable was the corrected children´s age. The dependent variable was the number of erupted teeth, by gender, evaluated according to some different outcome distributional forms. The generalized Akaike information criterion (GAIC) and the model residuals were used as the model selection criterion.

**Results:**

The Box-Cox Power Exponential method was the GAMLSS model with better-fit indexes. Our estimation curve was able to predict the number of erupted deciduous teeth by age, similar to the real values, in addition to describing the evolution of children’s development, with comparative patterns. There was no difference in the mean number of erupted teeth between the sexes. According to the reference curve, at 12 months old, 25% of children had four erupted teeth or less, while 75% had seven or fewer and 95% had 11 or fewer. At 24 months old, 5% had less than 12, and 75% had 18 or more. At 36 months old, around 50% of the population had deciduous dentition completed (20 teeth).

**Conclusion:**

The adjusted age was an important predictor of the number of erupted deciduous teeth. This outcome can be a variable incorporated into children’s growth and development curves, such as weight and height curves for age to help dentists and physicians in the monitoring the children’s health.

## Background

The dental eruption consists of a series of events in which the tooth migrates from its intraosseous position in the maxilla and mandible to its functional position. It culminates with an eruption in the gingival tissue and oral cavity. Therefore, it constitutes the last stage of the physiological process of a series of movements that the teeth execute, from the beginning of the odontogenesis to the end of the physiological cycle. However, most authors consider dental eruption only as the moment when teeth appear on the surface of the oral cavity, wich means the moment of dental emergence [1–4].

This process is part of a set of children´s somatic growth phenomena influenced by age, so dental age refers to the morphological state of an individual’s actual age [5]. Counts of emerged teeth may be used to make age estimations in children, to estimate accurately the age of those subjects who lack valid identity documents, and to study biological correlations of human developmental aspects. Because of this, age-related reference ranges are useful for assessing the number of erupted teeth in children [5, 6].

Logan´s human dental eruption chronology table, modified by McCall and Schour [7], is still accepted worldwide as a standard for a long time, although its findings were obtained, a long time ago, from the dissection of a few children´s cadavers from a specific population (European) and through a methodology that has not been well described. Other researchers have pointed out that the eruption occurred at least two months later in low-income countries [8–15], indicating that the origin, ethnicity, among other individual characteristics, can influence the chronology [2, 3, 13–21]. So, these variations should be considered in the development of a new standard for tooth eruption patterns, that cannot be universally applied owing to ethnic variations. Therefore, it would be more appropriate if these standards were obtained from the population to which will be applied.

Statistical techniques for the construction of age-related mathematical models have developed a lot in the last ten years, and this has been an area of particular interest in several research centers around the world [4, 22, 23].

As part of a broad consultative process to select the best statistical methods for the construction of new reference curves, in 2003, the World Health Organization (WHO) convened a group of experts, who recommended the use of the Box-Cox Power Exponential (BCPE) method with smoothing curves using cubic splines. This method is part of a wide class of statistical models called GAMLSS (Generalized Additive Models for Location, Scale, and Shape) [17], which consists of a regression theory for variables dependent on the exponential family of distributions. In this context, the aim of this study was to predict the deciduous teeth eruption in a Brazilian infant population from 12 to 36 months of age using the GAMLSS technique.

## Methods

### Study design

This is a cross-sectional study nested in the prospective cohorts (Etiologic factors of preterm birth and consequences of perinatal factors on child health: birth cohorts in two Brazilian cities – BRISA study) [24, 25], with children aged from 12 to 36 months, from two Brazilian cities: São Luís-MA and Ribeirao Preto-SP.

### Study location

São Luís-MA and Ribeirão Preto-SP are two Brazilian cities with contrasting socioeconomic situations. São Luís is a capital located on an island in the Northeast region, the poorest in the country. Its Human Development Index (HDI) was 0.768 in 2010, ranking 249th in Brazil. Per capita income was approximately $10,475.35 (2010), while the illiteracy rate for people aged 15 and over was 4.7%.

Ribeirão Preto, on the other hand, is located in the Southeast region of the country. The HDI was 0.8 in 2010, ranking 40th in Brazil. Per capita income was approximately $16,817.82 in 2010, while the illiteracy rate for people aged 15 and over was 2.9%, much lower than in São Luís.

### Study sample

**Fig. 1:**
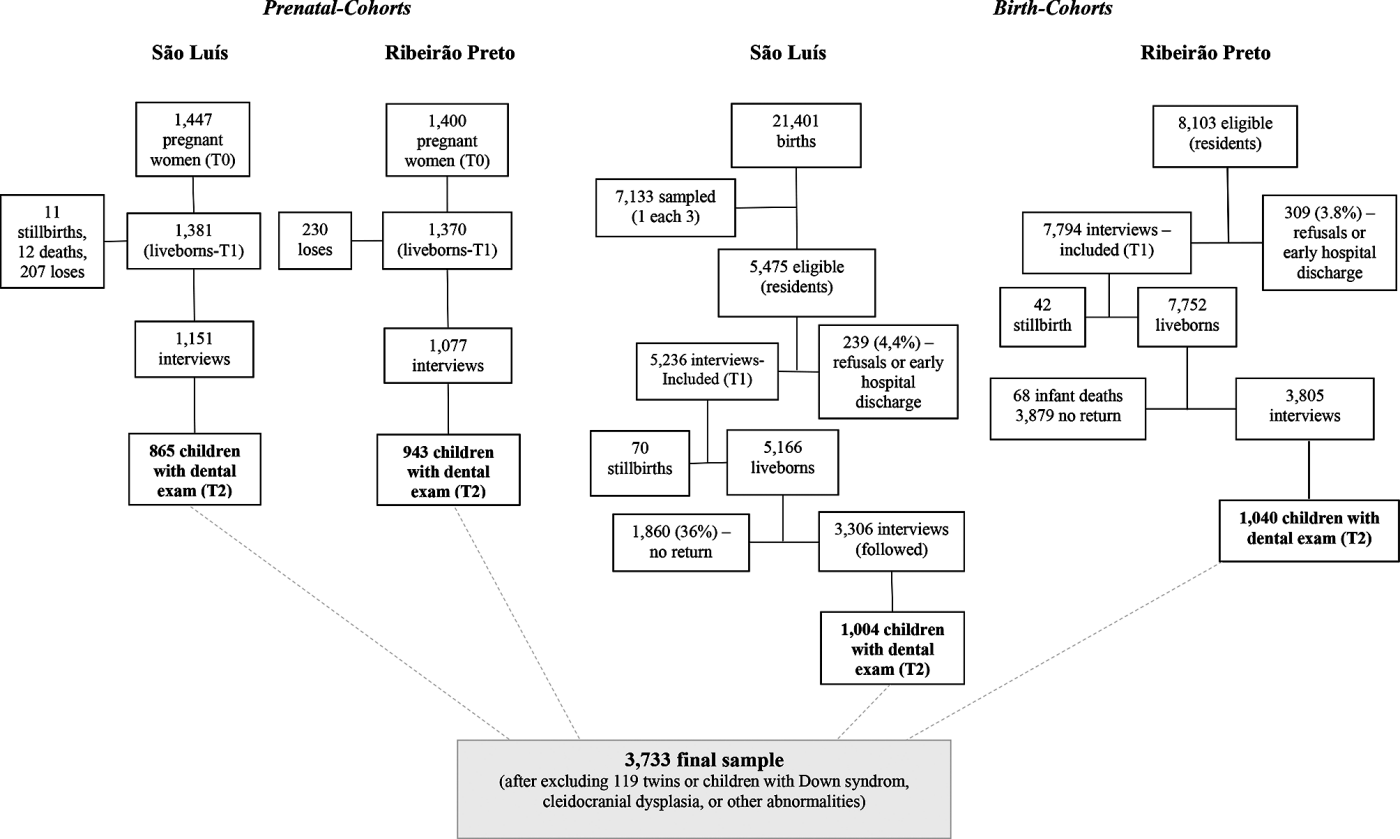
Flowchart of the study sample

BRISA cohort study is composed of four different cohorts: two (one in São Luís and another in Ribeirão Preto) initiated during prenatal (*PN-Cohorts*) and two (in the same places) initiated during the children´s birth (*Birth-Cohorts*). People included in the *PN-Cohorts* had assessments during prenatal care (T0) in 2010–2011, birth (T1) in 2010–2011, and children´s second years of life (T2) in 2011–2013. People at the *Birth-Cohorts* had assessments at T1 in 2010 and T2 in 2011–2013. The sample flowchart for the four different cohorts is summarized in Fig. 1.

The *PN-Cohorts* were composed of a convenience sample. In São Luís, we recruited women from three public maternities and one health center. A total of 1,447 pregnant women participated in T0. After losses, there were 1,381 for T1 and 1,151 children for T2. We performed dental examinations on *865 children*. In Ribeirão Preto, we recruited women from a register of pregnant women. The sample consisted of 1,400 pregnant women (T0). Of these, 1,370 (96.7%) were reevaluated in T1, while 1,077 returned with their children in T2 and *943 children* underwent dental examination (T3). In the present investigation, we have used only data from the T2 moment of the cohorts.

The *Birth-Cohorts* were designed as a stratified probabilistic sample with a systematic draw in São Luís. We set the minimum sample size at 6,000 births. There were 21,401 births during the year of 2010. After applying the sampling strategy, there were 5,475 eligible children. The final sample consisted of 5,236 postpartum women (T1) and their children in 2010. After the exclusion of 70 stillbirths, the final sample of this study was 5,166 births. We followed 3,306 infants and selected *1,004 children* for the dental examination (T2) in 2011–2013. In Ribeirao Preto, all births that occurred in eight institutions were included, totaling 8,103 births. Three hundred and night (3.8%) women did not agree to participate or were discharged early and were considered losses. So, there were 7,794 mothers interviewed in T1. In T2, we reassessed 3,805 children. Of these, *1,040 children* performed a dental examination. After excluding 119 people due to multiple gestations, Down syndrome, cleidocranial dysplasia, or other abnormalities, the final sample for this study was composed of 3,733 children who had dental examinations at T2 in both cities and cohorts.

### Variables and data collection

A trained team consisting of interviewers, examiners (dental surgeons), and assessors collected data. There were also field supervisors and study coordinators. The training, lasting 40 h, was theoretical and practical. Only examiners with inter- and intra-examiner kappa greater than 0.8 were selected for the team. Furthermore, a pilot was carried out for one full day to identify unforeseen situations and adapt workflows.

We used the following data collection techniques: (i) face-to-face interviews with children’s mothers, using semi-structured questionnaires, to collect the variables *sex* (dichotomous categorical variable) and *age* (continuous variable); and (ii) dental clinical exam of babies to collect the variable *number of erupted teeth* (continuous variable). The child’s age was corrected by the gestational age at birth. Gestational age was calculated through an algorithm based on two criteria: from the ultrasound performed in the first trimester of gestation and the date of the last menstrual period. The dental examinations were carried out with babies lying on their mothers’ laps in dental chairs in reserved rooms at the Federal University of Maranhão and the University of São Paulo. Five trained dentists in São Luís and two in Ribeirão Preto collected data under artificial light, after drying teeth with air blows, using a children´s mouth mirror. The tooth was considered presented when any part of the dental crown was visible on the surface.

### Statistical analysis

We estimated means and standard deviations for the number of erupted teeth using the software Stata, version 14.0. We used GAMLSS to model the distribution of erupted deciduous teeth as a smooth function of a single explanatory variable (corrected age). The GAMLSS model allows the creation of centile curves that vary as a function of the explanatory variable [22]. It is a generalization of the LMS method [22], a standard procedure for pediatric reference curves.

Given the explanatory variable, the response variable *Y* is modeled as a random variable with a density function *D*(*µ, σ, ν, τ* ), where each parameter (µ, σ, τ, ν) of the distribution response variable (number of deciduous teeth erupted) can be modeled as a function of explanatory variables.

$$\begin{aligned}& Y \sim D(\mu ,\sigma ,\nu ,\tau ) \hfill \\ & {g_1}\left( \mu \right) = {h_1}\left( x \right) \hfill \\ & {g_2}(\sigma ) = {h_2}\left( x \right) \hfill \\ & {g_3}(\nu ) = {h_3}\left( x \right) \hfill \\ & {g_4}\left( \mu \right) = {h_4}\left( x \right) \hfill \\ & x = ag{e^\xi } \hfill \end{aligned}$$

Where *µ, σ, ν*, and *τ* respectively represent the median, variability, skewness, and kurtosis parameters of the distribution. The *g*() functions represent appropriate link functions, the *h*() are non-parametric smoothing functions and ξ is a power transformation of age.

The relationship between these four parameters and age can take different forms. A wide variety of distributional forms are available to the outcome *Y* [26]. We used three distributions as possible models to represent the deciduous eruption: Box-Cox Cole & Green distribution (BCCG), Box-Cox-t (BCT), and Box-Cox Power Exponential (BCPE). We considered penalized B-splines (P-splines) to the mean (median) *µ*, variability *σ*, skewness *ν*, and kurtosis τ [22]. GAMLSS was fitted using the package *gamlss* in R [27, 28].

We used the generalized Akaike information criterion (GAIC) as a model selection criterion. The GAIC for a given model is GAIC(*b*) = − 2*L*(*µ, σ*, *ν*, *τ* ) + *b*. *Edf* with *b* > 0, *L* is the fitted log-likelihood function, and *edf* is the total effective degrees of freedom for a given model. A choice of *b* = 2 is equivalent to using Akaike Information Criterion (AIC), and *b* = *log*(*n*) with *n* equal to the number of observations is equivalent to the Bayesian Information Criterion (BIC). We selected the model with the lowest criterion value [22].

Once a GAMLSS model was fitted, we assessed the adequacy of the fitted model by examining the model residuals. Worm plots and the Q-Statistics [27] indicated the residual diagnostics. The worm plot is a de-trended normal Q-Q plot of the residuals. The model was inappropriate when many points plotted lie outside the (dotted) point-wise 95% coi nfidence bands [26].

### Ethic aspects

The Ethics Committees of the Hospital das Clínicas of the University of São Paulo in Ribeirão Preto (n. 4116/2008) and the University Hospital of the Federal University of Maranhão in São Luís (n. 350/08) approved the BRISA cohort study. The adults responsible for the children who were evaluated provided informed consent for participation in the research.

## Results

Table 1 shows the frequency of children with each erupted element and the mean of erupted teeth by gender in São Luís and Ribeirão Preto, from 2013 to 2015. There was a significant statistical difference between boys and girls only for three of the twenty deciduous teeth (elements 51, 52, and 54). But, in general, there was no difference, according to gender, in the mean of the number of erupted teeth (12.08% *versus* 12.01%; p-value = 0.87).

**Table 1:** Frequency of children with each erupted element and mean of erupted teeth by sex. São Luís and Ribeirão Preto, 2013–2015

	Boys (%) (n = 1896)	Girls (%) (n = 1826)	p-value
Element 51	99.1	98.23	0.02
Element 61	96.97	96.30	0.25
Element 71	81.36	83.58	0.07
Element 81	85.2	87.57	0.07
Element 52	93.93	92.15	0.04
Element 62	79.30	78.72	0.66
Element 72	77.91	79.10	0.37
Element 82	88.21	86.91	0.29
Element 53	45.5	42.7	0.09
Element 63	48.35	47.23	0.49
Element 73	6.56	58.15	0.24
Element 83	47.49	46.97	0.78
Element 54	62.91	66.50	0.02
Element 64	77.10	78.02	0.46
Element 74	72.17	72.07	0.95
Element 84	69.72	71.66	0.25
Element 55	37.92	35.30	0.14
Element 65	32.60	30.98	0.35
Element 75	16.23	16.17	0.96
Element 85	12	13.02	0.49
	$\mathop x\limits^ -$	$\mathop x\limits^ -$	p
Number of erupted teeth	12.08	12.10	0.87

$$\mathop x\limits^ -$$: sample mean

**Fig. 2:**
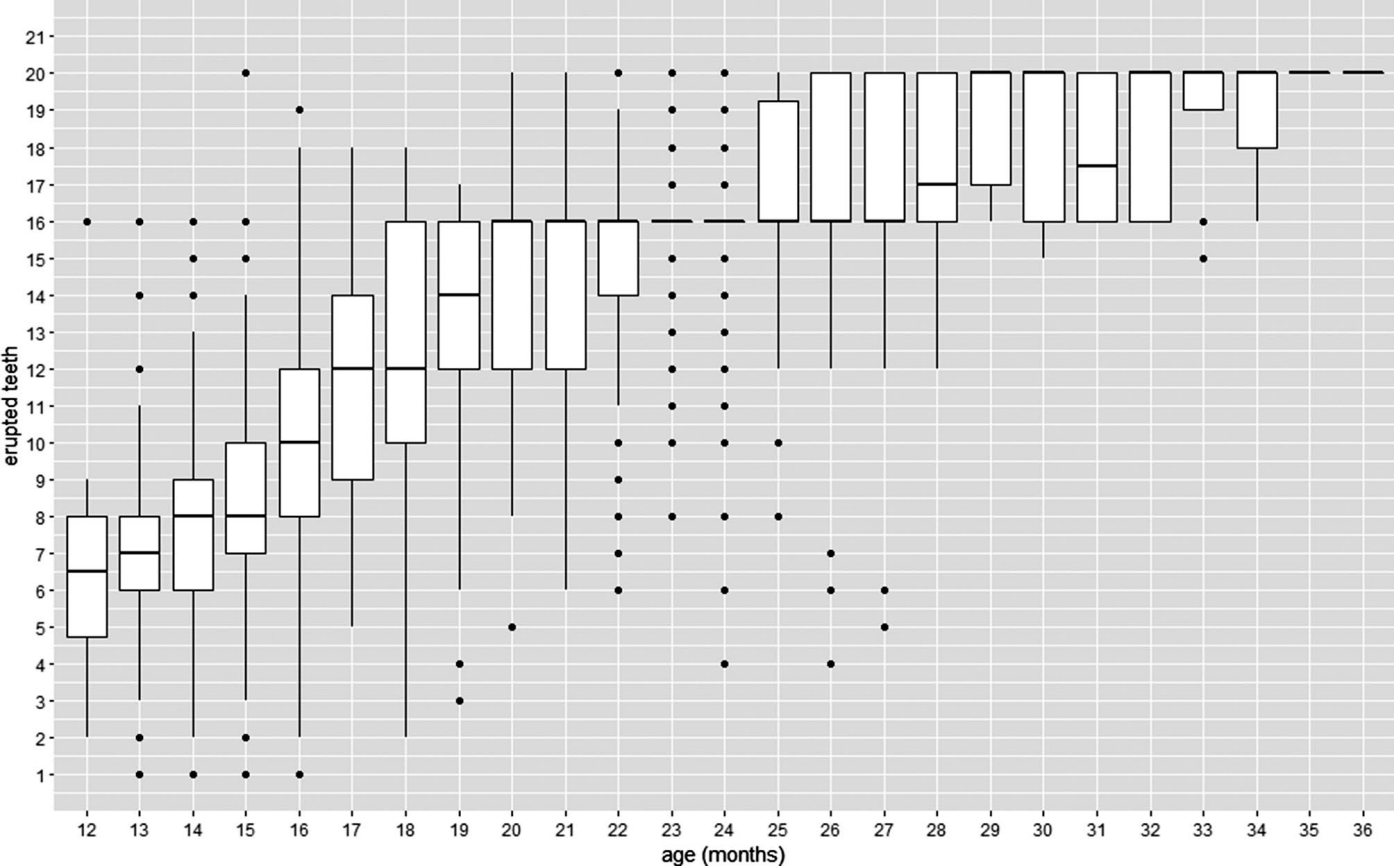
Box-plot graph for the number of erupted teeth by age

Figure 2 illustrates the descriptive statistics for the number of erupted teeth by corrected age in this sample. There was a high variability in the number of erupted teeth. At the age of 14 months, for example, the median number of erupted teeth was 8. The minimum value was 6, and the maximum value was 17. At the age of 30 months, the median was approximately 18, and at 36 months, everyone had the dentition completed (20 teeth).

The value of GAIC estimated from the models for all three distributions were: BCCG (19345.32), BCT (19345.68), and BCPE (19344.16). The model GAMLSS with BCPE distribution presented the smallest GAIC.

**Fig. 3:**
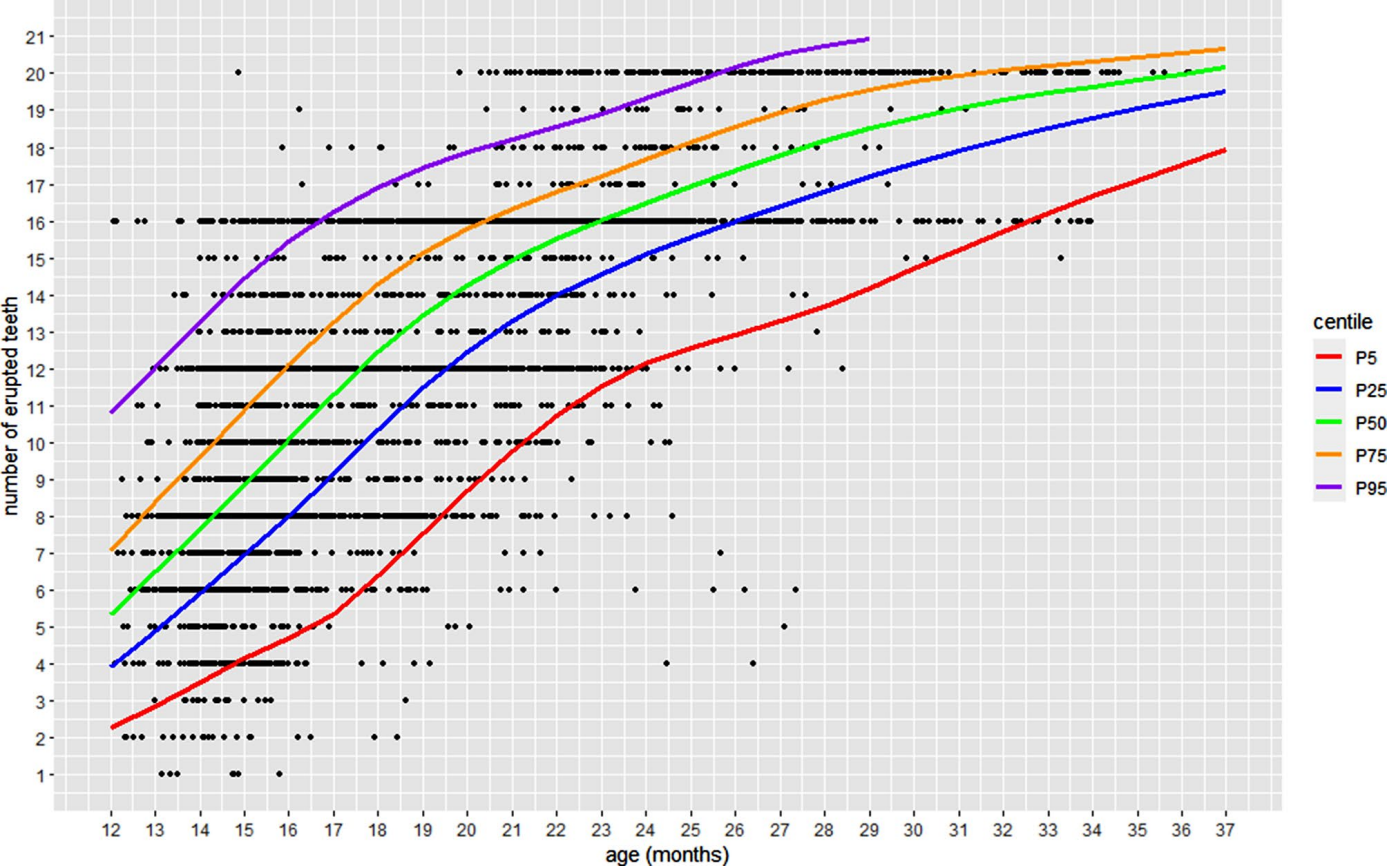
A Centile curve of the number of erupted deciduous teeth in a Brazilian population, 2013–2014

Figure 3 presents the fitted centiles for the final model (BCPE distribution). It shows the percentiles (5, 25, 50, 75, and 95) of the distribution of the number of erupted teeth according to the children’s corrected age. Around 5% of 12-month-old children had approximately two erupted teeth or fewer, while 50% (the most common situation) had six erupted deciduous teeth. 95% had less than 11 teeth. At 15 months, 50% of children had approximately three or fewer erupted teeth, while 95% had eight or fewer.

The older the age, the greater the number of predicted erupted teeth, indicating a good fit for the model. At 30 months old, 75% of the children had less than 20 erupted teeth, while at 34 months half of the children (represented by the 50th percentile) had the completed erupted deciduous teeth (20 teeth) (Table 2).

**Table 2:** Estimation of the number of erupted deciduous teeth by centiles. São Luis and Ribeirao Preto – Brazil, 2013–2014

Corrected age (months)	The mean number of erupted teeth by centile				
	5	25	50	75	95
12	2	4	5	7	11
13	3	5	6	8	12
14	3	6	8	10	13
15	4	7	9	11	14
16	5	8	10	12	15
17	5	9	11	13	16
18	6	10	12	14	17
19	7	11	13	15	17
20	9	12	14	16	18
21	10	13	15	16	18
22	11	140	15	17	18
23	11	15	16	17	19
24	12	15	16	18	19
25	13	15	17	19	20
26	13	16	17	19	20
27	13	16	17	18	20
28	14	17	18	19	20
29	14	17	18	19	20
30	15	18	19	20	20
31	15	18	19	20	20
32	16	18	19	20	20
33	16	18	19	20	20
34	17	19	20	20	20
35	17	19	20	20	20
36	17	19	20	20	20

***Prenatal-Cohorts Birth-Cohorts***

**São Luís Ribeirão Preto São Luís Ribeirão Preto**

## Discussion

By using an empirical model to evaluate several individuals of different ages, in a similar way to the growth reference curve, our model was able to predict the number of erupted deciduous teeth by age, generating a reference curve based on a local large sample of Brazilian children. In this statistical summary of data from a referential group of individuals, it is possible to verify how typical the measure of that individual is [4]. Very atypical patterns of eruption may indicate disturbances in growth and child development [29].

This information could be certainly important in reducing the concerns of families and healthcare professionals about the dental eruption of children because the classically used reference tables are based on older works with smaller samples and mainly with the European population. Studies conducted in Brazil [8–12] indicate that the eruption occurs at least two months later than the table of dental development by Logan and Kronfeld [7], slightly modified by McCall and Schour [30], which is classically used as a reference in books and at pediatric dentistry offices.

In these publications, anthropometric measures are assumed to have normal distribution. However, a lot of these variables present asymmetric distributions and, sometimes, kurtosis. The effect of the fourth moment of distribution (kurtosis) is meaningful in the estimate of the extreme percentiles, as, for example, the 3rd, 5th, 95th, and 97th percentiles [31].

Besides, usually, child growth curves are separated for boys and girls. However, in this work, we showed that there is no difference in the number of erupted elements between boys and girls. In most current works, this difference is not found [17, 32–36]. In the study conducted on Jordanian children (2018) [37], none of the teeth showed statistically significant differences in eruption chronology between genders. The child’s gender was also not associated with dental emergence patterns in a study conducted at Pelotas [32]. However, all incisors and the first upper molars erupted significantly earlier in boys in a study conducted in Polish children [35] and almost all deciduous teeth emerged earlier in boys in Spanish children [14]. In Pakistan [2], girls get early deciduous dentition than boys. These two studies evaluated the eruption of each tooth separately. On the other hand, Zhang et al. (2019) [20] found that the eruption age of the first deciduous teeth was earlier in boys than in girls, but they did not further evaluate the other erupted deciduous teeth.

Studies about chronology can describe the timing that the first tooth [38] or even each tooth erupts [8, 9] by follow-up studies, usually more viable in smaller sample, or describing the number of erupted teeth by a cross-sectional study [3, 10, 14–17, 20, 33, 35, 37] or estimating it by a probit analysis [13, 19, 34] also in a cross-sectional study, but none of these studies estimated possible values for the number of teeth per age in a centile curve, which considers different possible variations within a population, and allows the classification of how typical the number of erupted teeth for each child is. There are few studi’es of deciduous teeth eruption chronology in Brazil. Most aim to analyze factors associated with this phenomenon. Perhaps because of the difficulty of periodic evaluations in such small children. Ferreira et al. (2015) [9] carried out a study in Vitoria-ES, where they evaluated the average eruption period of each dental element. Other studies were carried out in Bahia [11] and Santa Catarina [10]. These studies also presented the findings in the same way, which makes comparison difficult. Haddad et al. (2005) [12] also estimated the number of erupted teeth in 908 children in the state of São Paulo, and the findings were similar. However, in this research, we presented the centiles of the distribution, which allows a larger acceptable margin of reference.

The original Logan`s dental eruption chronology table suggests that deciduous maxillary central incisors would erupt between 6 and 10 months, while the mandibular central incisors would erupt between 5 and 8 months [7]. It means that around 10 months, children must have the 8 incisor erupted. In this estimation, half of the children with 10 months had 7 erupted teeth (50th centile). Therefore, the results are similar, but this study also considers individual variations between the extreme centiles.

The selection of the cutoff for normality depends on several factors, such as the degree of sensibility and specificity that one wishes to give to the diagnosis, which is a function of the general situation in the population. In Brazil, the Ministry of Health, such as WHO recommendations, consider children between the 3rd and 10th centile at risk, while those who are under the 3rd centile are considered abnormal, for the growth curves. Acceptable values ​​for a population are those between the 3rd and 97th percentiles, which correspond to 94% of the estimated population, reflecting the variability of genetic potential among healthy individuals.

Another important finding is that at 30 months of corrected age, 75% of children had the 20 teeth erupted, while at 34 months, 50% of the population had the deciduous dentition completed (the most typical value). Most of the studies in Brazil show that at around 30 months, deciduous dentition is completed [8, 11, 12], later than in the classical studies of Showr and Massler (1947) [30], which showed that it occurred between 20 and 24 months.

Children with the number of erupted teeth under the 3rd or 5th centiles could present some diseases, such as Down Syndrome [39], Cleidocranial Dysplasia [40], Hunter Syndrome, Gardner’s syndrome, Turner Syndrome [41, 42], abnormalities in somatic growth [33, 36, 40﻿] or impaired functioning of the thyroid and pituitary glands [43, 44]﻿. Children with the number of erupted teeth above the 97th percentile could have endocrine abnormalities that accelerate metabolism [29].

GAMLSS is a class of fairly flexible models because it allows — besides choosing from a wide range of distributions for the dependent variable — various connection functions for the effects of predictive variables over the dependent variable. This estimated function, called a smoother curve, can take up different forms since it does not present the rigid structure of a parametric function [22]. An advantage of the erupted teeth-for-age chart is that it is possible to describe the evolution of a child’s development with comparative patterns, different from the classical tables.

This study had some limitations, such as the minimum age of children, which is 12 months, because of the initial cohort design. Besides, at some ages there was a small sample size, however, our model was still able to accurately predict the number of erupted deciduous teeth. The cross-sectional design did not allow us to accurately assess the moment of eruption of each tooth per child over time and unfortunately we do not have information on when each tooth began the eruption process. Instead, we have the result (measured by the examiner) of which teeth were present in the mouth at different ages, from 12 to 36 months of age. This strategy has been adopted in other epidemiological studies with larger samples [3, 13, 14, 45﻿]. But, to our knowledge, this is the first study including thousands of children from two Brazilian municipalities with contrasting sociodemographic characteristics to predict the number of erupted teeth and create a reference curve. Another strength of our study was the correction of the child’s age for preterm birth. This procedure is recommended given that around 10–12% of births in Brazil are to children with less than 37 weeks of gestational age [46, 47] and some studies have shown a slight delay in the time of dental eruption in preterm children [38, 45, 48–51], which disappears when corrected for gestational age at birth [48, 51, 52].

## Conclusions

This estimation curve was able to predict the number of deciduous teeth erupted by age in Brazilian children, similar to real values, in addition to describing the evolution of child development, with comparative standards. It may assist pediatricians in the evaluation of children´s somatic growth, as well as the identification of adjacent pathologies that could be related to the delay of tooth eruption, such as Down syndrome, cleidocranial dysplasia, or endocrine abnormalities. The number of teeth erupted by age could be a variable incorporated into children’s growth and development curves, such as weight and height curves for age.

## Data Availability

The datasets of the current study are not publicly available due to the sensitive nature of questionnaire information for the study community but are available from the corresponding author upon reasonable request.
